# Use of CytoSorb© Hemoadsorption in Patients on Veno-Venous ECMO Support for Severe Acute Respiratory Distress Syndrome: A Systematic Review

**DOI:** 10.3390/jcm11205990

**Published:** 2022-10-11

**Authors:** Ali Akil, L. Christian Napp, Cristina Rao, Teresa Klaus, Joerg Scheier, Federico Pappalardo

**Affiliations:** 1 Department of Thoracic Surgery and Lung Support, Ibbenbueren General Hospital, 49477 Ibbenbueren, Germany; 2 Department of Cardiology and Angiology, Hannover Medical School, 30625 Hannover, Germany; 3 CytoSorbents Europe GmbH, 12587 Berlin, Germany; 4 Cardiothoracic and Vascular Anesthesia and Intensive Care, AO SS. Antonio e Biagio e Cesare Arrigo, 15100 Alessandria, Italy

**Keywords:** hemoadsorption, ARDS, lung failure, inflammation, CytoSorb, ECMO

## Abstract

Acute respiratory distress syndrome (ARDS) is associated with high morbidity and mortality. Adjunct hemoadsorption is increasingly utilized to target underlying hyperinflammation derived from ARDS. This article aims to review available data on the use of CytoSorb© therapy in combination with V-V ECMO in severe ARDS, and to assess the effects on inflammatory, laboratory and clinical parameters, as well as on patient outcomes. A systematic literature review was conducted and reported in compliance with principles derived from the Preferred Reporting Items for Systematic Reviews and Meta-Analyses (PRISMA) statement. When applicable, a before-and-after analysis for relevant biomarkers and clinical parameters was carried out. CytoSorb© use was associated with significant reductions in circulating levels of C-reactive protein and interleukin-6 (*p* = 0.039 and *p* = 0.049, respectively). Increases in PaO2/FiO2 reached significance as well (*p* = 0.028), while norepinephrine dosage reductions showed a non-significant trend (*p* = 0.067). Mortality rates in CytoSorb© patients tended to be lower than those of control groups of most included studies, which, however, were characterized by high heterogeneity and low power. In an exploratory analysis on 90-day mortality in COVID-19 patients supported with V-V ECMO, the therapy was associated with a significantly reduced risk of death. Based on the reviewed data, CytoSorb© therapy is able to reduce inflammation and potentially improves survival in ARDS patients treated with V-V ECMO. Early initiation of CytoSorb© in conjunction with ECMO might offer a new approach to enhance lung rest and promote recovery in patients with severe ARDS.

## 1. Introduction

Acute respiratory distress syndrome (ARDS) represents one of the greatest challenges in intensive care medicine and mortality remains high [1]. ARDS can be precipitated by a variety of underlying disorders which can cause direct or indirect pulmonary injury via a dysregulated systemic inflammatory response. Released cytokines such as interleukin (IL)-1, IL-6, IL-8, and tumor necrosis factor activate neutrophils in the lung and fuel the inflammatory cascade [2]. As with septic shock [3,4], hyperinflammation and elevated cytokines play a major role in both hemodynamic instability and altered capillary permeability. The latter is a hallmark of ARDS and causes alveolar edema and diffuse atelectasis, resulting in life-threatening hypoxemia [5]. During the Coronavirus Disease 2019 (COVID-19) pandemic, we have seen a new type of ARDS that, while falling under the Berlin definition, differs from “normal” ARDS with distinctive features such as frequently preserved compliance, despite severe hypoxemia and widespread coagulopathy [6,7,8].

**Figure 1:**
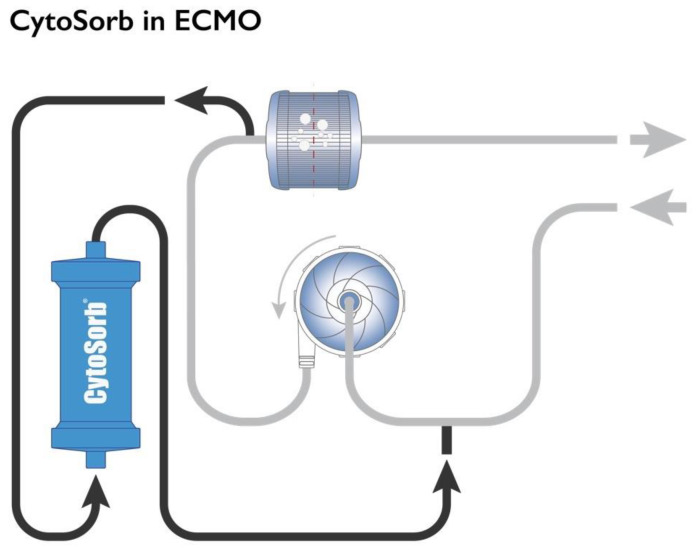
Integration of the CytoSorb© hemoadsorption in the ECMO circuit. Used with permission from CytoSorbents Europe GmbH.

The use of veno-venous extracorporeal membrane oxygenation (V-V ECMO) to resolve severe acute hypoxemia in severe cases of ARDS regardless of etiology has gained substantial interest over the last decade, but mortality in these patients still remains high [9]. Hemoadsorption with CytoSorb© (CytoSorbents, Princeton, NJ, USA) is increasingly utilized as an adjunct therapeutic option in this heterogenous and very sick patient population. The CytoSorb© whole blood adsorber is a CE-marked medical device. It can be integrated as a bypass circuit within the ECMO circuit itself (Figure 1) or can alternatively be inserted in concomitant continuous renal replacement therapy (CRRT) or hemoperfusion circuits. Of note, integration into the ECMO circuit typically leads to higher blood flow rates through the adsorber compared to hemoperfusion or CRRT circuits, and thus likely more effective substance clearance and a higher dose of hemoadsorption treatment [10].

Extracorporeal hemoadsorption attenuates an excessive systemic inflammatory response [11] by reducing circulating levels of inflammatory mediators, which may result in hemodynamic stabilization [12] and improved oxygenation [13]. In addition to cytokines, CytoSorb© adsorbs various pathogen associated molecular patterns (PAMPs), as well as damage-associated molecular patterns (DAMPs) [14], further downregulating immune activation [15].

Observational studies suggest that hemoadsorption facilitates faster hemodynamic stabilization and reduced need of vasopressors in patients with septic shock [16,17,18,19]. Early combined use of ECMO with CytoSorb© might reduce ventilator-induced injury by enhancing lung rest and at the same time treating the overshooting hyperinflammation and thus avoiding further deterioration of organ function [20]. Furthermore, data suggest that CytoSorb© use in combination with V-V ECMO may result in reduced SOFA scores already 24 h after start of CytoSorb treatment [13]. The effects on mortality in published reports vary: several data demonstrate lower observed versus predicted mortality [21,22,23,24], while studies showing higher mortality in CytoSorb©-treated patients have also been published [20]. Here, we aimed to analyze all available data to assess the effect of CytoSorb© adjunct therapy in patients with severe ARDS receiving V-V ECMO support.

## 2. Materials and Methods

This systematic review was conducted in compliance with the PRISMA Preferred Reporting Items Systematic Reviews and Meta-Analysis (PRISMA) guidelines [25] (see PRISMA 2020 checklist, Supplementary).

We performed a free-text terms literature search on PubMed using the search string (ECMO AND (hemoadsorption or Cytosorb)) for studies published from 2012 to today. Regardless of the study design, we retrieved full texts and abstracts of clinical studies. Studies had to be published in English language and conducted on patients treated concomitantly with CytoSorb© and V-V ECMO, irrespective of ARDS etiology or the type of circuit used for hemoadsorption. Studies were included into the final analysis if they reported at least on one of the following changes before and after treatment: inflammatory biomarker levels, including IL-6 (expressed as pg/mL), C-reactive protein (CRP) (mg/dL), procalcitonin (PCT) (ng/mL), D-dimer (mg/L) and ferritin (ng/mL), PaO2/FiO2 ratio (mmHg), norepinephrine dosage (µg/kgBW/min) and mortality. For biological markers and organ support parameters, all data that could be converted to the reference measurement scale were included. In order to account for the concentration-dependent adsorption rate of the device, only data from studies with baseline IL-6 levels equal to or higher than 150 pg/mL were considered [26]. Studies were excluded when the target patient population represented only a subgroup of the total sample, and when specific data for the ECMO subgroup could not be retrieved. Finally, the literature search was complemented by screening abstracts and articles submitted to or published in the context of relevant international conferences. Two authors (CR and TK) searched for and screened the literature independently. Controversies were solved with discussion and inclusion of a third author (JS).

A formal assessment of the risk of bias of included studies through available tools, such as the revised Risk of Bias tool (RoB 2) tool for randomized trials and the Risk Of Bias In Non-randomized Studies of Interventions (ROBINS-I) tool recommended by the Cochrane Library [27,28], was not possible due to the characteristics of most retrieved studies, which included non-interventional studies (i.e., case series, case reports) without distinct treatment groups. The risk of bias was assessed graphically through a funnel plot of the effects on the risk of mortality against standard errors.

We analyzed the potential effect of hemoadsorption on relevant parameters by conducting a before and after analysis using the paired-sample *t*-test [29]. Data were summarized as mean ± standard deviation. An exploratory analysis was conducted to assess the potential effect on mortality; we did so by comparing mortality observed in the CytoSorb© treated patients to mortality observed in control groups, wherever available. If no control group was available in the study, mortality as reported by the Extracorporeal Life Support Organization (ELSO) registry for COVID-19 patients, or, for studies not involving COVID-19 patients, mortality predicted by severity scores were used as controls. Specifically, a subgroup analysis was carried out to compare 90-day mortality observed in COVID-19 patients treated with CytoSorb© with mortality as expected based on geography-specific 90-day mortality reported in the ELSO registry for COVID-19 patients. The treatment effect on mortality was expressed as the mortality risk ratio of the treatment compared to the control group. All data were analyzed using Microsoft Excel version 16 (Microsoft Corporation. 2019. Redmond, WA, USA) and STATA statistical software, release 16 (StataCorp LLC. 2019. College Station, TX, USA) [30]. 

## 3. Results

**Figure 2:**
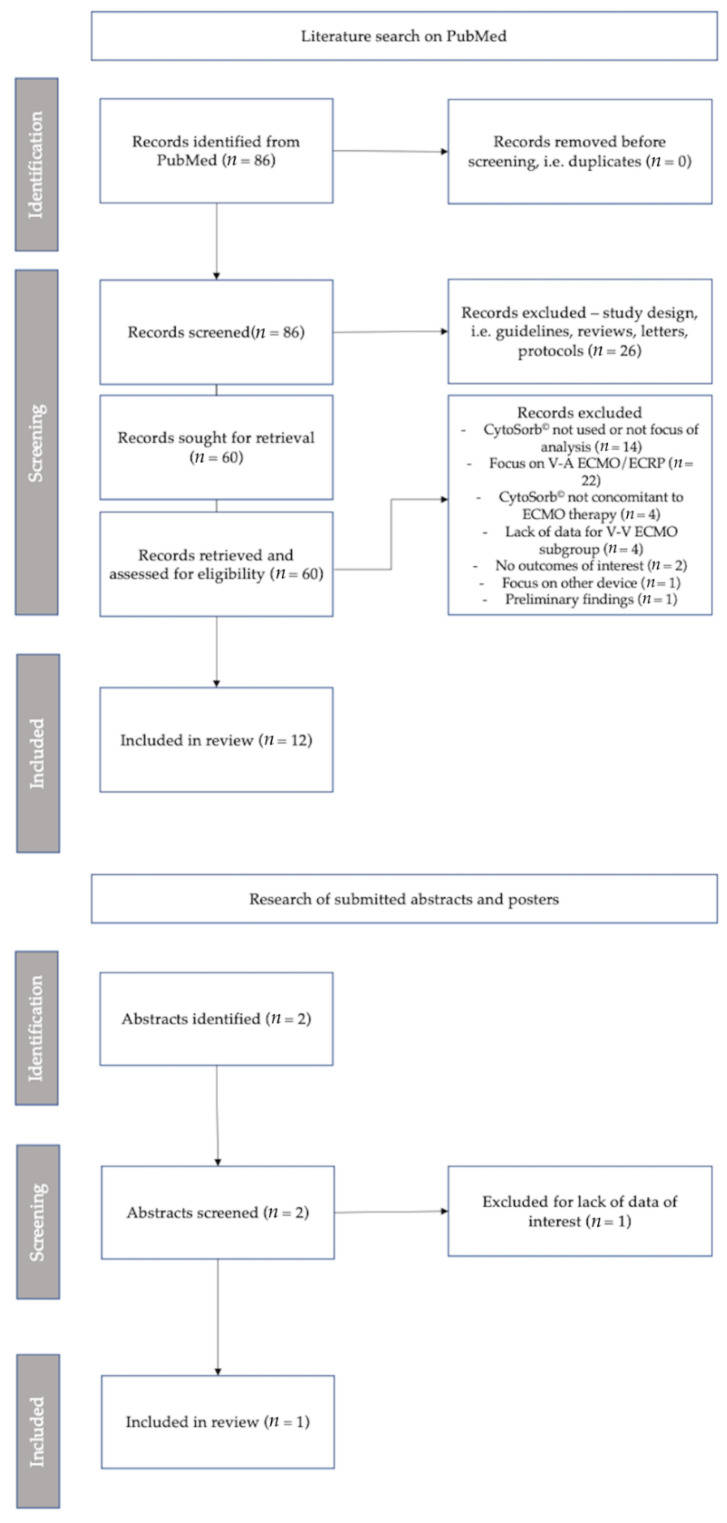
PRISMA flowchart of study screening and identification.

The literature search was conducted in PubMed on 14 June 2022. After excluding irrelevant articles (i.e., reviews, meta-analyses, protocols, letters), 60 studies were retrieved (Figure 2). Of these, 22 were excluded due to their focus on veno-arterial ECMO (V-A ECMO) or extracorporeal cardiopulmonary resuscitation (ECPR) [31,32,33,34,35,36,37,38,39,40,41,42,43,44,45,46,47,48,49,50,51,52]. Fourteen studies were excluded for lack or scarcity of data on CytoSorb© use [53,54,55,56,57,58,59,60,61,62,63,64,65,66], four because of the lack of specific data for the ECMO subgroup [67,68,69,70], four because CytoSorb© was not used concomitantly with ECMO therapy [71,72,73,74], two because outcomes of interest for the simultaneous use of CytoSorb© and ECMO were not reported [75,76], and one because it focused on a different device [77]. One study was excluded because it only reported preliminary findings from other articles [78]. In total, twelve studies were included at this stage. 

Finally, a dedicated search for presentations at international conferences that may not be published yet resulted in one abstract that was included for the analysis of mortality [79].

In summary, thirteen eligible studies [13,20,79,80,81,82,83,84,85,86,87,88,89] with sufficient data on the outcomes of interest were included (Figure 2). 

The key features of the studies are summarized in Table A1 in the Appendix A.

Most included studies consisted of observational studies; two prospective randomized trials with small sample sizes were also included [20,81]. 

**Figure 3:**
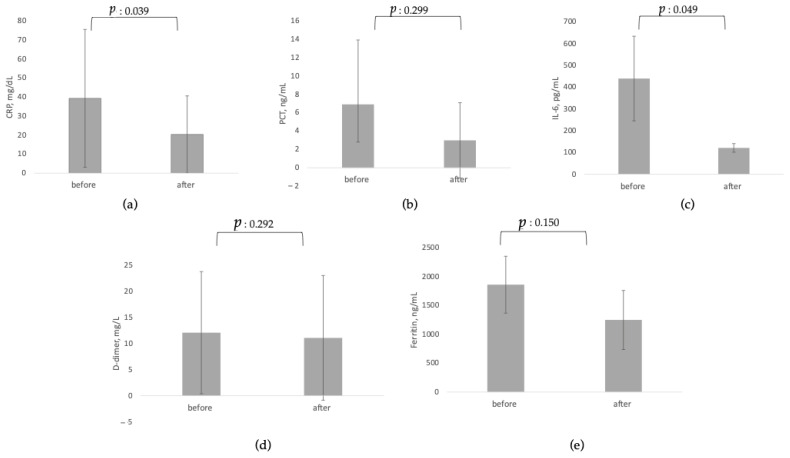
Before-and-after analysis for use of CytoSorb© on circulating biomarkers. (**a**) CRP, C-reactive protein; (**b**) PCT: procalcitonin; (**c**) IL-6, Interleukin 6; (**d**) D-dimer; (**e**) Ferritin.

**Figure 4:**
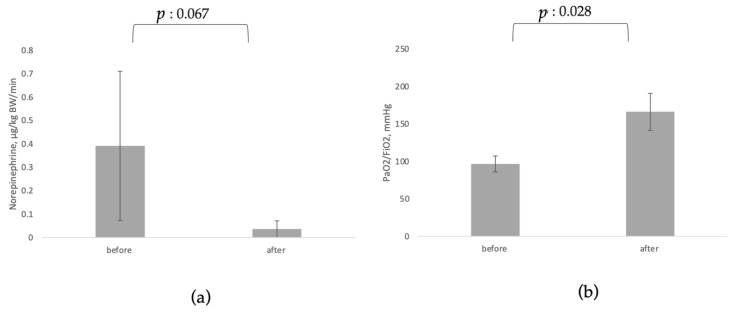
Before-and-after analysis of use of CytoSorb© on organ function. (**a**) Norepinephrine; (**b**) PaO_2_/FiO_2_.

Table 1 reports results of the before-and-after analysis for inflammatory markers and clinical parameters of interest, which are also presented graphically in Figure 3 and Figure 4.

### 3.1. Effects on Circulating Biomarkers, Organ Function and Organ Support

After the use of hemoadsorption, levels of all inflammatory markers were reduced from baseline. While reductions were most pronounced and reached statistical significance for CRP (*p* = 0.039) and IL-6 (*p* = 0.049), reductions in PCT, D-dimers and ferritin did not reach statistical significance. 

Data on vasopressor dosage before and after CytoSorb© treatment were available from three case series [80,82,88], a randomized controlled trial [20] and a case report [86]. There was a non-significant trend towards reduction in norepinephrine dosage (*p* = 0.067). Data on oxygenation requirements from three case series and a registry analysis [13,20,88,89] showed substantial and statistically significant increases in PaO_2_/FiO_2_ (*p* = 0.028). 

The study by Akil et al. showed shorter mean duration of V-V ECMO support in the CytoSorb© group compared with the control group (8.2 days, range 2–23 days vs. 19.3 days, range 13–30 days, *p*-value not available) [80]. In an exploratory analysis of the multicenter CTC registry on the use of CytoSorb© in COVID-19, two post hoc groups were created according to the median time to start of CytoSorb© after ICU admission, which was 87 h. A trend towards shorter ECMO duration was observed with earlier initiation of CytoSorb© following ICU admission [90]. 

### 3.2. Effect on Mortality

Whenever control data were not available, mortality observed in CytoSorb© patients was compared with mortality as recorded by the ELSO-registry for COVID-19 patients, or to mortality predicted by severity scores for non-COVID-19 patients (see Table 2), as stated in the methods section. As of May 2022, 90-day mortality was 49% in more than 8000 adult COVID-19 patients included from North America in the ELSO registry. In contrast, mortality was 30% at 30 days and 42% at 90 days in 2500 adult European COVID-19 patients [91].

In comparison to control groups or to predicted mortality as described above, CytoSorb© treatment was associated with lower mortality in 7 out of 10 studies (Table 3). 

**Figure 5:**
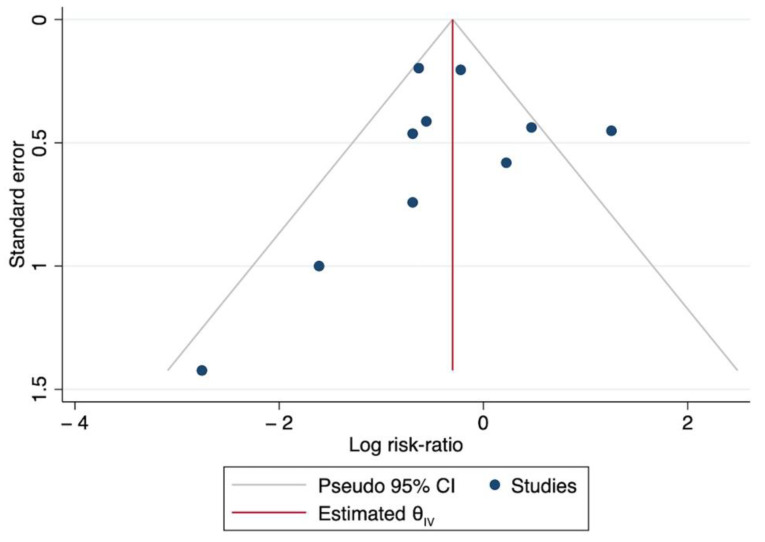
Funnel plot of the treatment effect on mortality against standard deviation for included studies.

The studies were highly heterogenous with regard to indication, study design and sample size. Neither of the two RCTs were adequately powered to detect any difference in mortality [20,81] and other articles consisted of non-interventional, retrospective studies. Figure 5 reports the funnel plot for the expected publication bias of the include studies. The asymmetry of the funnel plot of the log risk ratio of dying in the treatment group against their standard errors suggests a high level of bias exists within the studies. 

#### CytoSorb© and V-V ECMO in COVID-19 Patients

Acknowledging the limitations in terms of heterogeneity, small sample size and potential bias observed in the analysis above, we conducted an exploratory analysis of the treatment effect on mortality in the subgroup population of adult COVID-19 patients receiving V-V ECMO support. 

Out of the studies presented above, five articles [80,84,85,88,92] assess the effect on 90-day mortality of CytoSorb© as adjuvant therapy in the stated population. Observed results were compared to the “expected” mortality of 49% (for US studies) and 42% (for EU studies) reported in the ELSO registry (i.e., if these patients had followed the same course of disease as those included in the registry). The mortality rates from the ELSO registry were chosen as relevant historical control data, being ELSO the largest international registry on ECMO. Of note, we used the ELSO European mortality data to calculate mortality for the control group of studies conducted in Europe, specifically one study from the United Kingdom [85] and one from Germany [88]. In the patient population under question, for Germany higher mortality rates than in other countries have been observed and widely discussed [93,94,95], and this might be relevant and should be considered when appraising mortality data from studies conducted in Germany and the ELSO European mortality rate itself.

**Figure 6:**
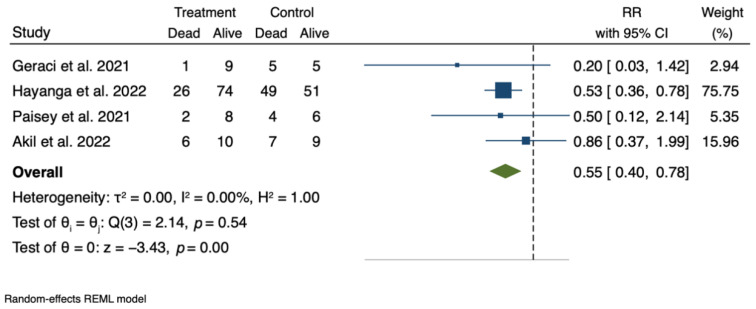
Pooled treatment effect of CytoSorb© therapy on 90-day mortality in COVID-19 patients receiving V-V ECMO support. RR: risk ratio; CI: confidence interval. See references [84,88,89,92].

The result of the pooled treatment effect is presented graphically in a forest plot (Figure 6). 

The analysis suggests that the treated patients might have a significantly lower risk of death compared to the control group (risk ratio, RR: 0.55. 95% CI: 0.40–0.78, *p* < 0.001). The data from the unpublished poster on the CTC registry have by far the largest impact and weight on the results of the analysis, which should be taken into account when considering and generalizing these results.

## 4. Discussion

In 1976, Dr. Robert Bartlett reported the first successful use of ECMO in the famous case of ‘Baby Esperanza’ [96]. Over the last 10 years, global ECMO use has increased significantly and is expected to further increase in the future, in light of improving clinical outcomes, increasing familiarity by institutions, and technological advances in ECMO circuits. This utilization trend may hold true especially for the management of novel respiratory viruses that are likely to emerge in the future [97]. However, it has also been suggested that the use of ECMO itself may evoke an inflammatory response [98]. Various mechanisms have been postulated as contributing to this process, including cellular activation, fibrinolysis, complement activation, secondary von Willebrand syndrome, hemolysis, molecules that are instigated by the surfaces of the circuit tubings, and the rotor/oxygenator, but also end-organ hyperperfusion/hyperoxygenation related to ECMO-derived non-pulsatile flow [99]. 

The current study reviewed the available evidence on the effects of adjunctive CytoSorb© therapy and V-V ECMO on several key inflammatory and clinical markers. The findings demonstrate that use of CytoSorb© therapy results in reduced levels of inflammatory and biological markers, presumably due to active removal by hemoadsorption (for IL-6 and PCT), or, as a secondary effect, due to improved inflammatory status. With regard to the latter, however, it is not clear to what extent the effect is directly and solely attributable to the hemoadsorption therapy. In addition, the mean baseline levels of CRP do not seem excessively high, which poses a necessary reflection on the extent of inflammation at baseline and the interpretation of the results in the different clinical conditions considered. 

Although not reaching statistical significance, reduction in ferritin was also observed which is in line with recent data on the use of CytoSorb© in COVID-19 patients [100]. Ferritin is involved in regulation of iron in the oxidative stress response [101] and a known predictor in the development of ARDS [102]. Furthermore, the therapy showed the potential to improve lung function and improve hemodynamic stability, with increased PaO_2_/FiO_2_ ratios and reduced vasopressor dosages. Of note, with regard to the respiratory function, it cannot be ascertained how much of the improved oxygenation should be ascribed to the effect of V-V ECMO itself. Likewise, when interpreting changes in vasopressor dose, details on fluid therapy and fluid balance would need to be considered, but this was not possible based on the data presented in the available publications. Although these findings are encouraging, proof that CytoSorb© improves survival is still preliminary. Among available datasets, five studies had control groups [20,80,81,82,88]. In one study on sepsis-associated ARDS, 13 patients in the CytoSorb© group had a survival rate of 100%, which endured through follow-up at 3–10 months, while 4 out of 7 patients (56%) in the control group died due to sepsis with multiorgan failure [80]. This exaggerated effect on mortality needs to be interpreted with caution, since it is derived from a small observational single-center study. However, it is generally consistent with the findings of another study [13] that showed a relative risk reduction of more than 50% when comparing observed (43%) with predicted mortality (91%), based on the Acute Physiology and Chronic Health Evaluation (APACHE) II score. Additionally, Simplified Acute Physiology Score (SAPS) II scores decreased significantly in the CytoSorb© group, which was not observed in the control group [80]. 

In the study by Rieder and colleagues, nine all-comers with severe ARDS predominantly from infectious causes, who were treated with V-V ECMO and cytokine adsorption, were compared with a control group of nine propensity-score-matched patients who had undergone V-V ECMO support without cytokine adsorption. Even though scores predicted a higher mortality in the hemoadsorption group, mortality was reduced in the CytoSorb© plus V-V ECMO group compared with V-V ECMO alone. In total, five patients in the CytoSorb© group survived (55.6%), compared withtwo2 (22.2%) from the matched control group. 

The single-center CYCOV randomized study reported that CytoSorb© therapy in combination with V-V ECMO was associated with higher mortality than ECMO alone in unselected COVID-19 patients [20]. Due to the small sample size (*n* = 34), the two randomized groups were severely imbalanced, including markedly higher D-dimer levels in the CytoSorb© group. Elevated D-Dimers suggest diffuse thrombotic microangiopathy with high thrombotic burden, causing diffuse ischemic organ injury and failure, and have been established as an independent marker of mortality in COVID-19 [103]. In addition, the study was powered for the primary endpoint of IL-6 reduction, which was negative, and not for clinical outcomes. The CYCOV study has been subject to controversial scientific discussions highlighting that—even in an RCT setup—uncertainties regarding equality of study groups as well as timing and dosing of hemoadsorption therapy should prevent any precipitous conclusions [104,105].

Recently, the multicenter CTC Registry reported high survival rates among 100 COVID-19 patients treated at five US centers under the FDA Emergency Use Authorization (EUA) (90-day mortality, 30%) [106]. 

Data have also shown shorter V-V ECMO support duration in patients treated with adjunctive CytoSorb© therapy [80]. Duration seems to be shorter when CytoSorb© is initiated earlier [90]. The potential to reduce ECMO duration could translate into significant economic benefits associated with the use of CytoSorb©, given the shorter ECMO duration and the high costs of V-V ECMO therapy in general.

This analysis summarizes the current status of published articles on patients treated with V-V ECMO and CytoSorb©; however, it has several limitations. First, due to the limited number of data available, studies were included without any consideration of the study design and characteristics or etiology of ARDS. Secondly, the overall number of patients observed is relatively small, while the heterogeneity and potential bias of studies is high. This should be carefully considered when appraising the findings from the pooled analysis. In addition, the study contributing the most to the pooled exploratory analysis was a registry-based one which not peer-review published yet. Finally, the magnitude of effects of the concomitant extracorporeal therapy itself (ECMO and CRRT) on the patient course could not be assessed in this study.

## 5. Conclusions

To the best of our knowledge, this is the first comprehensive summary of the available data on the clinical effects of combined CytoSorb© and V-V ECMO treatment. The safety and feasibility of the device have previously been demonstrated in multiple clinical scenarios with various technical setups. Despite low patient numbers, there was a trend towards effective inflammatory biomarker reduction, decreased vasopressor dosage and improved lung function with adjunctive hemoadsorption. Exploratory analyses suggest that the aforementioned clinical benefits may also translate into lower mortality. These results, although preliminary, warrant prospective controlled studies to further investigate the effect of CytoSorb© in patients on V-V ECMO for severe ARDS, in order to better characterize the clinical effects of this novel therapy in this very high-risk population. Combined and early use of extracorporeal membrane oxygenation and hemoadsorption could represent a novel strategy to promote enhanced lung rest in patients with ARDS.

## Figures and Tables

**Table 1:** Effect of CytoSorb© on parameters of interest.

	Before CytoSorb Mean ± SD	After CytoSorb Mean ± SD	*p*-Value	Patients *n*
CRP, mg/dL [80,83,84,85,86,87,89]	39.35 ± 36.2	20.39 ± 20.24	0.039	74
PCT, ng/mL [80,84,85,87]	6.90 ± 7.01	2.98 ± 4.10	0.299	36
IL-6, pg/mL [20,86,87,88]	439.50 ± 194.45	120.65 ± 19.72	0.049	39
D-dimer, mg/L [20,83,84,85,86,89]	12.07 ± 11.69	11.07 ± 11.94	0.292	70
Ferritin, ng/mL [84,85,86,87,89]	1860 ± 492.50	1249.12 ± 511.32	0.15	41
Norepinephrine, µg/kg BW/min [20,80,82,86,88]	0.391 ± 0.319	0.036 ± 0.035	0.067	56
PaO2/FiO2, mmHg [13,83,88,89]	96.55 ± 10.62	166.08 ± 24.66	0.028	59

CRP, C-reactive protein; PCT, procalcitonin; IL-6, interleukin 6; BW, body weight; SD, standard deviation. The paired-sample t-test for equality of the mean (±SD) was used considering the normal distribution of most variables included.

**Table 2:** Characteristics of studies reporting mortality of ARDS patients treated with CytoSorb© and V-V ECMO.

Study	Indication	Mortality Reported at	Control Group
Akil et al., 2021 [80]	ARDS; sepsis	30 days	Reported cohort
Supady et al., 2021 [20]	COVID-19	30 days	Reported cohort
Akil et al., 2022 [88]	COVID-19	90 days	Reported cohort
Stockmann et al., 2022 [81]	COVID-19	30 days	Reported cohort
Rieder et al., 2021 [82]	ARDS	ICU	Reported cohort
Hayanga et al., 2022 [79]	COVID-19	90 days	ELSO registry for COVID-19 in the US
Pieri et al., 2021 [83]	COVID-19	30 days	ELSO registry for COVID-19 in the EU
Geraci et al., 2021 [84]	COVID-19	90 days	ELSO registry for COVID-19 in the US
Paisey et al., 2021 [85]	COVID-19	90 days	ELSO registry for COVID-19 in the EU
Kogelmann et al., 2020 [13]	ARDS; sepsis	30-day; hospital	APACHE II

ARDS, Acute Respiratory Distress Syndrome; ELSO, Extracorporeal Life Support Organization; ICU, Intensive Care Unit; RCT, Randomized Controlled Trial.

**Table 3:** Mortality in ARDS patients treated with CytoSorb© and V-V ECMO vs. control or predicted mortality.

Study	Study Design	CytoSorb© Patients, *n*	Mortality %	Source of Control/Predicted Mortality	Control Patients *n*	Mortality %	∂ Mortality ARR
Akil et al., 2021 [80]	Retrospective, observational	13	0%	Control group	7	57%	−57%
Supady et al., 2021 [20] *	RCT	17	82%	Control group	17	24%	+58%
Akil et al., 2022 [88] *	Retrospective, observational	16	38%	Control group	10	30%	+8%
Stockmann et al., 2022 [81] *	RCT	9	78%	Control group	7	100%	−22%
Rieder et al., 2021 [82]	Retrospective, observational	9	44.4%	Control group	9	78%	−33%
Hayanga et al., 2022 [79] *	Retrospective, observational	100	26%	ELSO US registry	100	49%	−23%
Pieri et al., 2021 [83] *	Retrospective, observational	15	54%	ELSO EU registry	15	30%	+24%
Geraci et al., 2021 [84] *	Retrospective, observational	10	10%	ELSO US registry	10	49%	−39%
Paisey et al., 2021 [85]	Retrospective, observational	10	20%	ELSO EU registry	10	42%	−22%
Kogelmann et al., 2020 [13]	Retrospective, observational	7	43%	APACHE II (39)	7	91%	−48%

ARDS, acute respiratory distress syndrome; ARR, Absolute risk reduction; * Indicates studies on patients with COVID-19-related ARDS.

## Data Availability

Data used in the analysis are available upon request.

## Supplementary Materials

The following supporting information can be downloaded at: https://www.mdpi.com/article/10.3390/jcm11205990/s1, PRISMA 2020 Checklist.

## Appendix A

Details of studies included.

**Table A1:** Characteristics of studies included in the analysis. PCT: procalcitonin; CRP: C-reactive protein; IL-6: interleukin-6; ICU: intensive care until. Patients treated, *n*, include only patients in the study that have been treated with CytoSorb and V-V ECMO. For inflammatory markers, norepinephrine and PaO2/FiO2 ratio, only studies reporting on both before and after CytoSorb treatment are considered.

Reference	Indication	Patients Treated *n*	Controls *n*	Mortality Reported At	Inflammatory Markers	Norepinephrine Dosage Reported	PaO_2_/FiO_2_ Ratio Reported
Akil et al., Thorac Cardiovasc Surg 2021; 69(3):246–251 [80]	ARDS	13	7	30 days	PCT, CRP	✓	-
Song et al. Front Med 2021; 8:773461 [interim analysis] [89]	ARDS/COVID	52		ICU, 30 days, 90 days	CRP, ILs-6, D-dimer	-	✓
Kogelmann et al., J Intensive Care Society 2020: 21(2):183–190 [13]	ARDS	7		28 days, ICU and hospital		-	✓
Hayanga et al., 2022 Abstract No 000494, The European Society of Intensive Care Medicine (ESICM) 2022 [79]	ARDS/COVID	100		90 days	PCT, CRP, IL-6, D-dimer	-	(✓)
Geraci et al., J Cardiac Surg 2021; 36(11):4256–4264 [84]	ARDS/COVID	10		Overall	PCT, CRP, IL6, D-dimer	-	-
Pieri et al., Int J Artif Organs. 2022; 45(2):216–220 [83]	ARDS/COVID	15		ICU	CRP	-	✓
Supady et al., Lan Resp Med 2021; 9(7): 755–762 [20]	ARDS/COVID	17	17	30 days	IL-6, D-dimer	✓	-
Rieder et al., Artif Organs 2021; 45(2):191–194 [86]	ARDS/COVID	1		-	IL-6 D-dimer	✓	-
Rodeia et al., Blood Purif 2021; epub [87]	ARDS/COVID	5		Overall/unspecified	PCT, CRP, IL-6	-	-
Rieder et al., ASAIO J. 2021 Mar 1;67(3):332–338 [82]	ARDS	9	9	ICU	-	✓	-
Paisey et al., Int J Artif Organs. 2021 Oct;44(10):664–674 [85]	ARDS/COVID	10		ICU	PCT, CRP, IL-6, D-dimer, Ferritin	-	-
Stockmann et al., Crit Care Med. 2022;50(6):964–976 [81]	COVID	9	7	30 days	-	-	-
Akil et al., Int J Artif Organs 2022 Jul;45(7):615–622 [88]	ARDS/COVID	16	10	90 days	IL-6	✓	✓
